# Placebo Analgesia, Nocebo Hyperalgesia, and the Cardiovascular System: A Qualitative Systematic Review

**DOI:** 10.3389/fphys.2020.549807

**Published:** 2020-09-25

**Authors:** Hojjat Daniali, Magne Arve Flaten

**Affiliations:** Department of Psychology, Norwegian University of Science and Technology, Trondheim, Norway

**Keywords:** placebo effects, nocebo effects, cardiac activity, blood pressure, heart rate, heart rate variability, pain, review—systematic

## Abstract

**Background:** Placebo/nocebo effects involve the autonomic nervous system, including cardiac activity, but studies have reported inconsistent findings on how cardiac activity is modulated following a placebo/nocebo effect. However, no systematic review has been conducted to provide a clear picture of cardiac placebo responses.

**Objective:** The main goal of the present study is to review the effects of placebo analgesia and nocebo hyperalgesia on cardiac activity as measured by blood pressure, heart rate, and heart rate variability.

**Methods:** Using several Boolean keyword combinations, the PubMed, EMBASE, PsycINFO, Cochrane Review Library, and ISI Web of Knowledge databases were searched until January 5, 2020, to find studies that analyzed blood pressure, heart rate, or heart rate variability indexes following a placebo analgesic/nocebo hyperalgesic effect.

**Results:** Nineteen studies were found, with some reporting more than one index of cardiac activity; eight studies were on blood pressure, 14 studies on heart rate, and six on heart rate variability. No reliable association between placebo/nocebo effects and blood pressure or heart rate was found. However, placebo effects reduced, and nocebo effects increased low-frequency heart rate variability, and heart rate variability significantly predicted placebo effects in two studies.

**Conclusion:** Placebo/nocebo effects can have reliable effects on heart rate variability, but not on heart rate and blood pressure.

## Introduction

Placebo analgesia is a reduction in pain due to the administration of an inert substance with information that the substance effectively alleviates pain (Flaten et al., 2006; Benedetti, 2008). Moreover, inert factors previously associated with effective treatment, for example, through learning procedures such as classical (e.g., Ader, 1997; Flaten et al., 1997; Flaten, 2013), observational (e.g., Colloca and Benedetti, 2009; Hunter et al., 2014; and Bajcar and Babel, 2018), and operant conditioning (Adamczyk et al., 2019; Babel, 2020), are also capable of generating placebo effects. Both verbal information and learning procedures can generate expectations about positive outcomes, which are known as one of the mechanisms of placebo effects (Kirsch, 2004; Flaten et al., 2013). However, recent evidence suggests that conditioning procedures can be regarded as a distinctive mechanism of placebo effects, as placebo effects induced by conditioning are not always mediated via expectations (Babel et al., 2018; Babel, 2019). On the opposite side, negative expectations/experiences about a treatment, induced by either verbal suggestions (e.g., Stovner et al., 2008) or learning mechanisms (e.g., Bajcar et al., 2020), can lead to higher pain, anxiety, and physiological stress levels (e.g., Flaten et al., 1999, 2011; Aslaksen and Lyby, 2015; Roderigo et al., 2017). The body of research on nocebo effects is relatively small, however.

Placebo/nocebo responses impact various physiological processes, especially those controlled by the autonomic nervous system (ANS) (Meissner, 2014). The ANS controls visceral organs and tissues through sympathetic and parasympathetic nervous divisions to preserve homeostasis. Homeostasis is the ongoing process of maintaining physiological equilibrium, which comprises autonomic, neuroendocrine, and behavioral mechanisms (Craig, 2002; Meissner, 2014).

The cardiovascular system is under ANS control and has an essential role in the maintenance of homeostasis (Berntson et al., 2017). Pain, as a stressor, increases sympathetic activity (SA) (Craig, 2003; Loggia et al., 2011) and leads to corresponding changes in cardiac activity. In a systematic review (SR), Koenig et al. (2014) found 20 studies on experimentally induced pain that also reported subsequent cardiac activity. The authors concluded that following painful stimulation, cardiovascular SA increased and parasympathetic activity (PA) decreased (Koenig et al., 2014). Based on such studies, it can be assumed that if a placebo effect reduces pain, then it also reduces cardiac SA and increases cardiac PA. However, the use of various cardiac activity metrics makes it challenging to draw clear conclusions about cardiac placebo responses. Blood pressure (BP), heart rate (HR), and HR variability (HRV) (described in Materials and Methods) are three cardiac activity indexes that have been used to observe cardiac reactions to pain (for a review, see Koenig et al., 2014) and placebo effects (e.g., Amigo et al., 1993; Aslaksen and Flaten, 2008; Hrobjartsson and Gotzsche, 2010; Zimmermann-Viehoff et al., 2013).

That is why an SR is warranted to synthesize the findings on cardiovascular placebo analgesic/nocebo hyperalgesic responses. To our knowledge, this is the first SR aimed to unravel the modulatory effects of placebo analgesia and nocebo hyperalgesia on cardiac activity. To do so, the following questions were investigated: (a) Which cardiac metric, among BP, HR, and HRV, gives the best picture of cardiac activity following a placebo/nocebo effect on pain in adults? (b) Is a change in cardiac activity essential for a placebo/nocebo effect to occur on pain?

## Materials and Methods

### Search Strategy

The Boolean keyword combinations presented in Table 1 were used to search the PubMed, EMBASE, PsycINFO, Cochrane Review Library, and ISI Web of Knowledge databases until January 5, 2020. The systematic approach used for this SR is in accordance with the Preferred Reporting Items for Systematic Reviews and Meta-Analyses (PRISMA) statement (Moher et al., 2009).

**Table 1:** Boolean terms used to search databases.

	**OR**	**AND**	**AND**
“HRV”	“Heart rate variability”	“Placebo effect”	“Pain”
“HRV”	“Heart rate variability”	“Placebo response”	“Pain”
“HRV”	“Heart rate variability”	“Nocebo”	“Pain”
“HRV”	“Heart rate variability”	“Hypoalgesia”	“Pain”
“HRV”	“Heart rate variability”	“Hyperalgesia”	“Pain”
“HRV”	“Heart rate variability”	“Expectation”	“Pain”
“HRV”	“Heart rate variability”	“Expectancy”	“Pain”
“HR”	“Heart rate”	“Placebo effect”	“Pain”
“HR”	“Heart rate”	“Placebo response”	“Pain”
“HR”	“Heart rate”	“Nocebo”	“Pain”
“HR”	“Heart rate”	“Hypoalgesia”	“Pain”
“HR”	“Heart rate”	“Hyperalgesia”	“Pain”
“HR”	“Heart rate”	“Expectation”	“Pain”
“HR”	“Heart rate”	“Expectancy”	“Pain”
“BP”	“Blood pressure”	“Placebo effect”	“Pain”
“BP”	“Blood pressure”	“Placebo response”	“Pain”
“BP”	“Blood pressure”	“Nocebo”	“Pain”
“BP”	“Blood pressure”	“Hypoalgesia”	“Pain”
“BP”	“Blood pressure”	“Hyperalgesia”	“Pain”
“BP”	“Blood pressure”	“Expectation”	“Pain”
“BP”	“Blood pressure”	“Expectancy”	“Pain”

### Data Extraction

The first author extracted the data, and both authors reviewed them. The searches resulted in 2,613 hits, which were reduced to 1,806 after duplicates were removed. In this SR, the main target outcomes were BP, HR, or HRV indexes of cardiac activity following a placebo/nocebo effect, as compared to the control or a comparison group/condition. The secondary target outcomes were pain reports (e.g., pain intensity, threshold, duration, tolerance, unpleasantness, symptom severity, pain treatment efficacy, and pain expectation). Therefore, only studies in English that reported either BP and/or HR and/or HRV following a placebo/nocebo effect on pain (either experimental or clinical) were included.

**Figure 1:**
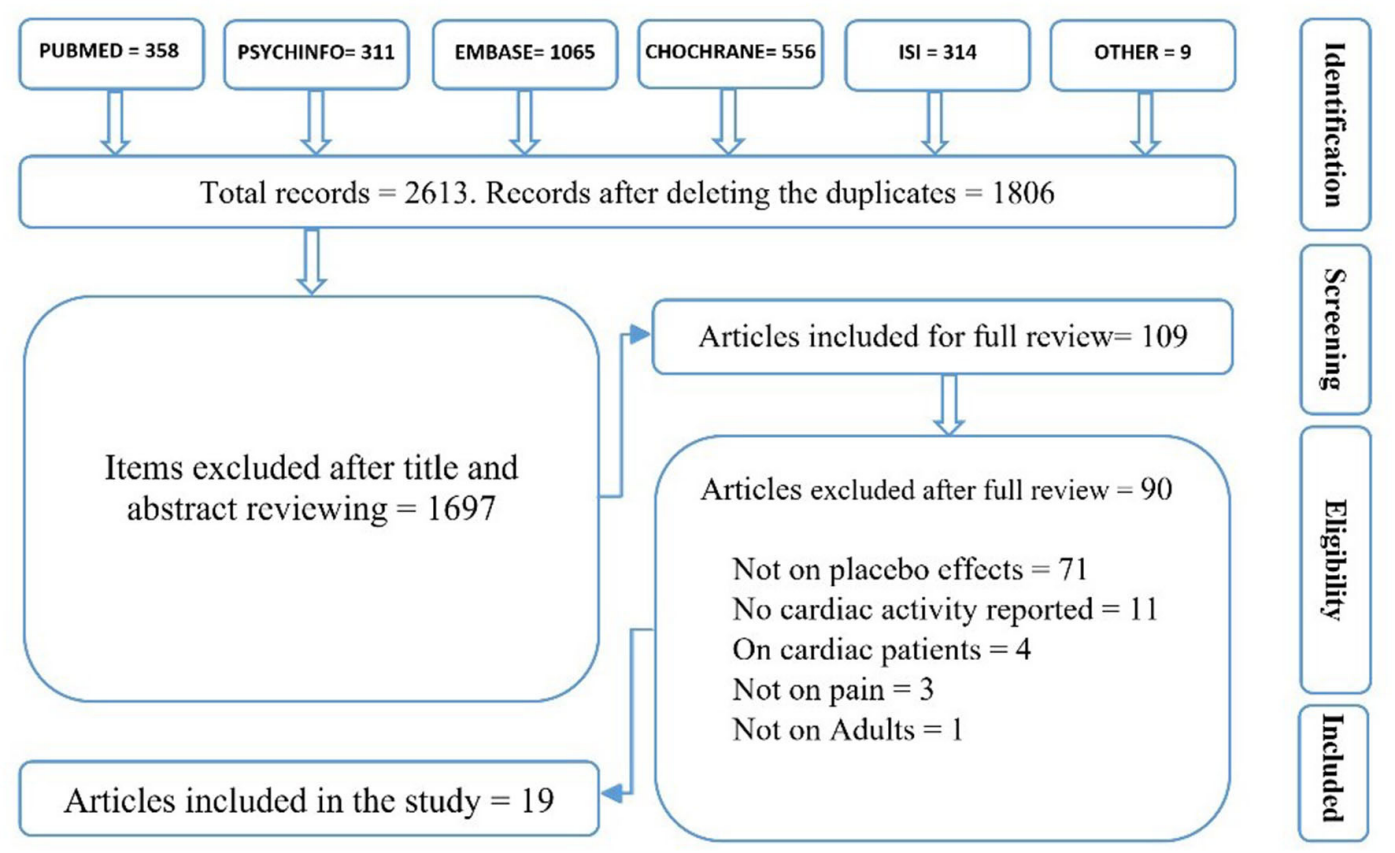
The review flow diagram.

A placebo effect was defined as a reduction in pain due to information that a treatment/manipulation would reduce pain, compared to the natural history control group/condition or the group/condition with different manipulation. The same definition was applied for a nocebo effect, except that instead of the reduction in pain, an increase in pain was assumed (Flaten et al., 2006). Either of the following situations was considered as a cardiac placebo response: a change in BP, HR, or HRV metrics following the administration of a placebo/nocebo treatment/condition compared to the control or the comparison group/condition (Meissner, 2014). Studies on both healthy subjects and patients experiencing pain were included. However, due to potential differences in cardiac activity (e.g., faster HR in children or irregular HR in cardiac patients), studies on children, animals, and cardiac patient populations were excluded. After reviewing the titles and abstracts, 109 relevant articles were identified for a thorough article review. The same inclusion criteria as in sorting out the hits were applied to read the articles: only (a) peer-reviewed studies (b) in English on (c) human adults and on (d) pain (either experimental or clinical) which (e) have investigated cardiac activity following a placebo/nocebo effect with (f) at least two comparison groups/conditions or a control group/condition (e.g., a natural history control group or a control group/condition) were included. After review of the 109 articles, 19 were included in the present SR (Figure 1).

Although this SR does not have a protocol review, there is a list of excluded studies, accessible by contacting the first author (HD) (Figure 1).

### Cardiac Measurement Indexes

#### BP

BP is the pressure of blood flowing in vessels, expressed through systolic BP (SBP; the maximum pressure through one heartbeat) and diastolic BP (DBP; the minimum pressure between two beats) (Berntson et al., 2017). BP is controlled through the baroreceptor reflex of afferent nerves placed in the aortic and carotid artery walls that signal acute stretches in the artery walls or pressure changes to the nucleus tractus solitarius (NTS) in the medulla. NTS provides a negative feedback (i.e., baroreflex activity), meaning that in response to a baroreflex signal of an elevation in BP, NTS increases PA, which will result in HR and BP decreases (France and Ditto, 1996). This relationship implies that decreases in BP are associated with elevated PA, whereas increases in BP are associated with elevated SA (Sved, 2009).

#### HR

HR is computed by counting the number of heart contractions over a period of time, usually 1 min. The heart is mostly under inhibitory influence of PA (Thayer, 2009). SA increases lead to HR increases, and increases in PA result in HR decreases. The basic data to compute HR are the time interval between two adjacent beats (R spikes), the interbeat interval (IBI) (Thayer, 2009).

#### HRV

The parasympathetic and sympathetic branches both modulate cardiac activity, and HRV is computed to extract and separate these influences (Koenig et al., 2014; Berntson et al., 2017). There are three main methods to calculate HRV: time domain, frequency domain, and non-linear domain. However, the basic data to compute all types of HRV are the IBI. Time domain measures compute HRV in short-term (minutes or seconds) to long-term (hours) recordings, although short-term recordings are more common in pain and placebo studies (e.g., Aslaksen and Flaten, 2008; Koenig et al., 2014). The main method to quantify HRV from time domain metrics is R–R intervals, which is the calculation of the time interval between two R spikes in an electrocardiogram. R–R intervals are also called normal-to-normal (N–N) intervals when it is assured that the R spikes are intact and not affected by artifacts (Citi et al., 2012). Common statistical methods to analyze HRV are the standard deviation of N–N intervals (SDNN) and the root mean square of successive differences between normal R spikes (RMSSD). SDNN represents autonomic effects on a 5-min HRV recording (Minarini, 2020) and represents PA influences (Shaffer et al., 2014). RMSSD is obtained by computing successive time differences between R spikes. Then, each value is squared and averaged before obtaining the root for the total value. RMSSD is primarily used to measure changes in HRV that are mediated by the vagal nerve (Task force of the European society of cardiology and the north American society of pacing electrophysiology, 1996). Frequency domain methods use power spectral analysis to calculate a certain set of frequency bands in the IBI. Two frequency bands are usually discernable from short-term recordings: the high-frequency (HF; 0.15–0.4 Hz) band that is primarily mediated by PA and represents the respiratory influences and also a combination of SA and vagal activity. The HF band is highly correlated with respiratory sinus arrhythmia (RSA; i.e., heart period changes due to the respiratory cycles) and RMSSD and is related to vagal effects (Koenig et al., 2014). The low-frequency (LF; 0.04–0.15 Hz) band is indicative of PA and baroreflex activity (Thayer et al., 2008; Goldstein et al., 2011) and can be derived from short timescale recordings. To assess pain-related HRV, both time and frequency domain indices are used. The HF/LF ratio is another measure that represents the sympathovagal balance (Thayer, 2009). Non-linear methods analyze the irregularity and complexity of the cardiac time series data (Thayer, 2009), are little used, and will not be assessed here.

### Bias Risk Assessment and Grading the Quality of Evidence

Biased reporting (i.e., changing the study aims based on findings) and selection bias (i.e., lack of access to all available data) are two potential sources of bias that threaten the reliability of SRs (Drucker et al., 2016). To avoid biased reporting and selection bias, the aims of the study did not change throughout the project, and the reference and citation lists of all included studies were manually searched (Drucker et al., 2016). Moreover, the revised version of the *Cochrane Risk of Bias Tool* (RoB 2) was used to assess the risk of bias in included studies. RoB 2 assesses level of risk of bias (high, low, or some concerns) based on the randomization process (i.e., biases in randomization and random assignment), deviations from intended interventions (i.e., differences between the intervention and the comparison/control group), missing outcome data, measurement of the outcome (e.g., the objectiveness of the outcome assessment), and selection of the reported result (i.e., considering both reported and unreported data) (for more information, see Sterne et al., 2019). To evaluate the certainty of evidence found in this SR, the Grades of Recommendation, Assessment, Development, and Evaluation (GRADE) approach was used. GRADE classifies the equality of evidence as high (i.e., there is little chance that further research will change the confidence in evidence), moderate (i.e., future research is likely to impact the confidence in evidence), low (i.e., future research is very likely to impact the confidence in evidence), and very low (i.e., the evidence is very uncertain) based on the studies' design, inconsistency of the results, indirectness of the evidence, imprecision of the results, and publication bias (for more information, see Brozek et al., 2009).

## Results

A total of 19 studies were included. Eight studies reported more than one cardiac measurement; hence, eight studies reported BP, 14 studies reported HR, and six studies reported HRV. Included studies were classified based on the author names and publication year, sample size, study design, sample type, pain type and test, placebo/nocebo effects, and cardiac placebo responses (Table 2).

**Table 2:** Characteristics of studies investigating placebo/nocebo responses on blood pressure, heart rate, and heart rate variability.

**References**	**N (F)**	**Study Design. Groups/conditions**	**Sample. Pain induction method/pain type. Pain test**	**Pain-related placebo/nocebo responses**	**Cardiac placebo responses**
Robertson et al. (1991)	60 (30)	Between subjects. Three groups: (1) A treatment group who watched a stress reduction program. (2) A placebo group who watched a sham program. (3) A control group	Dental surgery patients. Dental surgery pain. Pin	Females in the placebo group had lower pain intensity compared to the treatment and control groups	No placebo response on BP. Females in the placebo group had lower HR
Geers et al. (2010)	116 (60)	Between subjects. Two groups: (1) A placebo group in which a placebo cream was introduced as a painkiller. (2) A control group in which the cream was introduced as a hand cleanser	Healthy participants. Cold pressor. Pin, MPQ^*^	Participants with higher optimism in the placebo group had lower pain intensity	No placebo response on BP and HR
Aslaksen et al. (2014)	75 (37)	Mixed design. Three groups: (1) A treatment group who received active treatment. (2) A placebo group who received minimal treatment. (3) A control group with no treatment	Healthy participants. Heat pain. Pin	The placebo group had lower pain intensity than the control group	The placebo group had lower SBP than the control group
Aslaksen et al. (2015)	142 (73)	Mixed design. Six groups: (1) Analgesic cream plus placebo information. (2) Analgesic cream plus nocebo information. (3) Placebo cream plus placebo information. (4) Placebo cream plus nocebo information. (5) Analgesic cream with no information. (6) Control with no treatment	Healthy participants. Heat pain. Pin	Nocebo information induced higher pain intensity regardless of whether the analgesic or the placebo cream was administered	Nocebo information induced higher SBP compared to the treatment or the placebo groups
Geers et al. (2015)	134 (66)	Between subjects. Two groups: (1) A placebo group in which a placebo cream was introduced as a painkiller. (2) A control group in which the cream was introduced as a hand cleanser	Healthy participants. Cold pressor. Pin, MPQ	The placebo group had lower pain intensity as compared to the control group	Participants with no prior pain experience had lower SBP in the placebo group. No placebo responses on HR
Roderigo et al. (2017)	120 (60)	Mixed design. Six groups: (1) High stress plus placebo information. (2) High stress plus neutral information. (3) High stress plus nocebo information. (4) No stress plus placebo information. (5) No stress plus neutral information. (6) No stress plus nocebo information	Healthy participants. Rectal distention visceral pain. Pin	Compared to neutral information, placebo information reduced pain, and nocebo information increased pain only in the stressed group	No placebo/nocebo responses on BP and HR. No effect of sex on HR and BP
Elsenbruch et al. (2019)	120 (60)	Mixed design. Six groups: (1) Relaxation plus placebo information. (2) Relaxation plus nocebo information. (3) Relaxation plus neutral information. (4) Control plus placebo information. (5) Control plus nocebo information. (6) Control plus neutral information	Healthy participants. Rectal distention visceral pain. Pin, Pex, Pun	Positive information decreased pain intensity and unpleasantness, compared with the control group. Positive information reduced pain expectation, and negative information increased pain expectation	SBP and HR decreased in the relaxation-plus-placebo information group
Pollo et al. (2003)	37 (30)	Between subjects. Two groups: (1) A placebo group who received a placebo pill plus placebo information. (2) A control group who received no treatment.	Patients seeking autonomic function assessment. Electric shocks. Pin.	Placebo group had lower pain than the no-treatment group	Placebo response reduced HR
	58 (31)	Second sample: Mixed design. Four groups which were each tested twice with hidden and open injections of(1) saline, (2) naloxone, (3) atropine, and (4) propranolol	Healthy participants. Tonic noxious stimulation. Pin	Placebo condition (saline) had lower pain than the hidden injection of the naloxone	Placebo response reduced HR. Placebo decreased LF components of HRV
Nishikawa et al. (2005)	104 (55)	Between subjects. Four groups: (1) Lorazepam plus complete information. (2) Lorazepam plus minimal information. (3) Placebo plus complete information. (4) Placebo plus minimal information	Dental patients. State anxiety. Dental pain. Pain facial scale	Patients who received complete information in both lorazepam and placebo groups had lower pain and anxiety than the minimal-information groups	There was lower HR in the placebo plus complete information than in the placebo plus minimal information group
Colloca and Benedetti (2009)	48 (48)	Between subjects. Three groups: (1) Placebo through social observation learning. (2) Placebo through conditioning. (3) Placebo through verbal suggestion	Healthy participants. Electric shock. Pin	Participants in the social observation and the conditioning groups had lower pain intensity than the verbal information group	No placebo response on HR
Bjørkedal and Flaten (2012)	72 (36)	Mixed design. Three groups (placebo information, nocebo information, and no information) × two conditions (conditioned pain stimulation and no conditioned pain stimulation)	Healthy participants. Heat pain. Pin, Pun	Positive information elicited lower pain, and negative information elicited higher pain intensity only in females	No placebo/nocebo response on HR. No effect of sex on HR
Jegindø et al. (2013)	40 (26)	Mixed design. Two groups (religious vs nonreligious) × three conditions (prayer to God, secular prayer, and no-prayer control)	Healthy participants. Electric shock. Pin, Pun	Prayer reduced pain intensity and unpleasantness in religious participants.	No effects of beliefs on BP and HRV
Benedetti et al. (2015)	35 (10)	Mixed design. Five groups (control, oxygen, placebo, placebo + conditioning, conditioning) × five testings	Healthy participants. Headache pain. Pin, fatigue	Sham oxygen plus conditioning decreased fatigue and headache as compared to the control group	Placebo plus conditioning decreased HR
Peerdeman et al. (2015)	116 (61)	Mixed design. Two groups (placebo information and neutral information) × two conditions (imagining best health condition and imagining typical health condition)	Healthy participants. Cold pressor. Pin	Placebo information and positive imagery induced positive expectations. No effects on pain	No placebo response on HR
Rhudy et al. (2018)	133 (68)	Between subjects. Four groups: (1) Expectation only. (2) Conditioning only. (3) Expectation + conditioning. (4) Control	Healthy participants. Electric shock. Pin	Expectation plus conditioning lowered pain intensity	No placebo response on HR acceleration
Aslaksen and Flaten (2008)	63 (32)	Within subjects. Two conditions: (1) Placebo pill. (2) Control	Healthy participants. Heat pain. Pin, Pun	Placebo capsules induced lower pain intensity than the control condition	No placebo response on HR. Lower LF/HF ratio in the placebo condition. HRV predicted a placebo effect on subjective stress
Chae et al. (2008)^†^	17 (0)	Between subjects. Two groups: (1) Positive information about acupuncture. (2) Negative information about acupuncture	Healthy participants. Acupuncture pain sensitivity. Pin, valence and arousal	Negative information resulted in lower valence and higher arousal scores than positive information	Negative information increased LF-HRV. Negative information did not change LF/HF ratio
De Pascalis and Scacchia (2019)	65 (65)	Within subjects. Two conditions: (1) Placebo cream plus positive suggestions. (2) Pain condition	Healthy participants. Cold pain. Pin, Pex, Pth	Placebo condition reduced pain as compared to the pain condition	Linear HRV predicted the placebo effect. The placebo increased the time-HRV. No placebo response on nonlinear HRV or LF/HF ratio
Adler-Neal et al. (2019)	62 (31)	Between subjects. Two groups: (1) Mindfulness treatment. (2) Sham mindfulness treatment	Healthy participants. Heat pain. Pin, Pun	Both interventions reduced pain intensity	Lower HF-HRV in the sham mindfulness group

N, sample size; F, females; Pth, pain threshold; Pto, pain tolerance; Pin, pain intensity; Pex, pain expectation; Pun, pain unpleasantness;

*, sensory and affective subscales;, this study had two samples;, none of the patients had any chronic pain or any abnormal autonomic nervous functioning;

†, *this study is included since different types of information about the same treatment was given to participants*.

### BP and Placebo/Nocebo Responses

A total of eight studies investigated BP following placebo/nocebo responses.

Four studies showed that participants had lower SBP in the placebo group and higher SBP in the nocebo group: Aslaksen et al. (2014) showed that exposure to a sham treatment reduced pain and SBP compared with the control or treatment groups. Geers et al. (2015) showed that participants without experience with a pain task in the placebo group had lower pain and SBP as compared with the control group. Elsenbruch et al. (2019) reported that positive suggestions plus a relaxation program lowered pain and SBP as compared with the control or negative information group. On the other hand, Aslaksen et al. (2015) showed that compared with the control group, nocebo information induced higher pain and SBP regardless of whether an analgesic cream or a placebo was administered. However, in four other studies, the placebo/nocebo effect had no impact on BP (Robertson et al., 1991; Geers et al., 2010; Jegindø et al., 2013; Roderigo et al., 2017) (Table 2).

To sum up, four studies showed that the placebo effect was associated with reduced SBP (Aslaksen et al., 2014; Geers et al., 2015; Elsenbruch et al., 2019) and that the nocebo effect was associated with increased SBP (Aslaksen et al., 2015). However, four other studies reported that the placebo/nocebo effects were not associated with a significant change in BP (Robertson et al., 1991; Geers et al., 2010; Jegindø et al., 2013; Roderigo et al., 2017) (Table 2).

Therefore, no reliable association is found between the placebo/nocebo effect and BP.

### HR and Placebo/Nocebo Responses

HR placebo responses were investigated in 14 studies (the study by Pollo et al., 2003, is counted twice, as it investigated HR in two different samples).

Six studies showed that the placebo effect reduced HR: Robertson et al. (1991) showed that females in the placebo group had lower pain and HR, as compared with the treatment and control groups. Pollo et al. (2003) studied two different samples and reported lower HR in the placebo group/conditions in both samples, as compared with the control group/conditions. Nishikawa et al. (2005) showed that regardless of the treatment type, adequate information lowered pain, anxiety, and HR, as compared with the minimal-information groups. Benedetti et al. (2015) showed that a conditioned sham treatment lowered headache, fatigue, and HR, as compared with the control group. Elsenbruch et al. (2019) showed that positive suggestions plus relaxation lowered pain and HR as compared with the control group. However, eight other studies reported that placebo/nocebo effects had no impact on HR (Aslaksen and Flaten, 2008; Colloca and Benedetti, 2009; Geers et al., 2010, 2015; Bjørkedal and Flaten, 2012; Peerdeman et al., 2015; Roderigo et al., 2017; Rhudy et al., 2018) (Table 2).

In sum, six studies showed that placebo effects reduced HR (Robertson et al., 1991; Pollo et al., 2003; Nishikawa et al., 2005; Benedetti et al., 2015; Elsenbruch et al., 2019), and eight studies showed that placebo effects did not impact HR (Aslaksen and Flaten, 2008; Colloca and Benedetti, 2009; Geers et al., 2010, 2015; Bjørkedal and Flaten, 2012; Peerdeman et al., 2015; Roderigo et al., 2017; Rhudy et al., 2018). No nocebo effects on HR was reported (Table 2).

Thus, the results do not suggest a reliable association between the placebo effect and HR.

### HRV and Placebo/Nocebo Responses

A total of six studies investigated HRV and placebo.

All six studies used power spectral analysis including time and frequency domains. Moreover, three studies analyzed the LF/HF ratio (Aslaksen and Flaten, 2008; Chae et al., 2008; De Pascalis and Scacchia, 2019); one study considered the coefficient component variance in LF and HF bands (Jegindø et al., 2013); and another study analyzed only HF-HRV (Adler-Neal et al., 2019).

Pollo et al. (2003) showed a decrease in LF-HRV in the placebo condition, as compared with the treatment conditions. Chae et al. (2008) showed that compared with positive information, negative information increased LF-HRV; notably, negative information had no effect on the LF/HF ratio. Aslaksen and Flaten (2008) showed that although the placebo effect did not impact HRV frequency, it lowered the LF/HF ratio. Moreover, HRV predicted a placebo effect on negative emotions. De Pascalis and Scacchia (2019) reported lower pain and increased time-HRV with a slowed-down pace in the placebo condition, as compared with the pain-only condition. Furthermore, although the LF/HF ratio was not impacted, linear HRV measures predicted the placebo effect. Adler-Neal et al. (2019) reported that the sham meditation program lowered pain and HF-HRV (only HF was analyzed). Finally, Jegindø et al. (2013) reported that religious beliefs did not impact HRV components (Table 2).

In sum, two studies showed that placebo effects decreased LF-HRV (Pollo et al., 2003) and that nocebo information increased LF-HRV (Chae et al., 2008), whereas one study showed that expectations did not affect HRV (Jegindø et al., 2013). Two studies showed that HRV predicted placebo responses on negative emotions and pain (Aslaksen and Flaten, 2008; De Pascalis and Scacchia, 2019). One study showed that sham meditation produced lower pain and HF-HRV (Adler-Neal et al., 2019). One study reported a lower LF/HF ratio following the placebo effect (Aslaksen and Flaten, 2008), but two studies showed the placebo effect failed to impact the LF/HF ratio (Chae et al., 2008; De Pascalis and Scacchia, 2019) (Table 2).

The results indicate that the placebo analgesic effect is associated with a decrease in LF-HRV, that the nocebo hyperalgesic effect is associated with an increase in LF-HRV, and that HRV is a predictor for placebo effects. However, there is no reliable effect of placebo on the LF/HF ratio and HF-HRV.

### Results of Bias Risk Assessment and Grading the Quality of Evidence

Of the 19 included studies, 14 had a low risk of bias in the randomization process, 13 had a low risk of bias in deviation from intended interventions, 18 had a low risk of bias in missing outcome data, 16 had a low risk of bias in measurement of the outcome, and 16 had a low risk of bias in selection of reported results. Overall, 12 studies were judged as having a low risk of bias (Table 3).

**Table 3:** Cochrane Risk of Bias tool for the included studies.

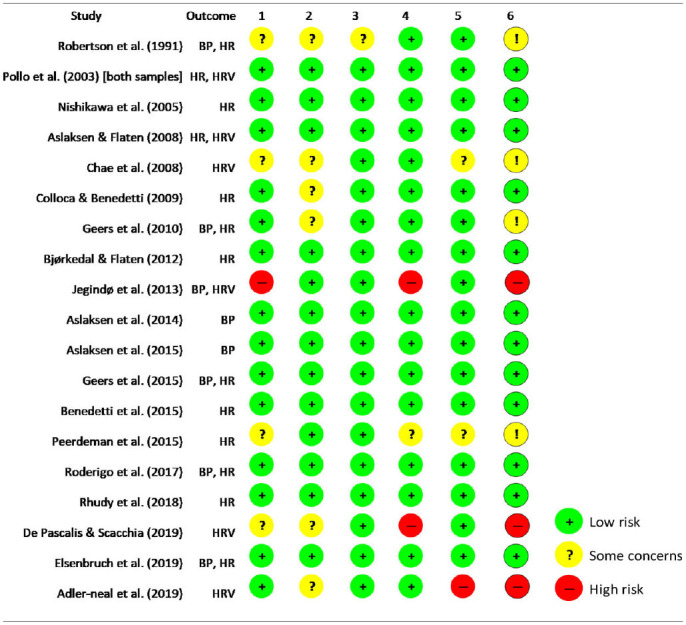

Numbers' definition.

1. Randomization process.

2. Deviation from intended interventions.

3. Missing outcome data.

4. Measurement of the outcome.

5. Selection of the reported result.

*6. Overall Bias*.

As presented in Table 4, the first and second findings (i.e., the BP and HR are not reliable cardiac indexes for the assessment of placebo/nocebo cardiac responses) are evaluated as having high certainty. The third finding (i.e., the HRV is a reliable cardiac metric for the assessment of cardiac placebo/nocebo responses), on the other hand, is judged as having low certainty, mainly due to heterogeneity of studies and manipulations, selective reporting, lack of natural history control groups, small sample size, and various statistical methods being used in some of the included studies.

**Table 4:** Grading the certainty of findings.

**Finding**	**Studies**	**Risk of bias**	**Inconsistency**	**Indirectness**	**Imprecision**	**Publication bias**	**Overall certainty of evidence**
Placebo/nocebo effects have no reliable effect on BP	8 randomized trials	not serious	not serious	not serious	not serious	none	⊕⊕⊕⊕ HIGH
Placebo/nocebo effects have no reliable effect on HR	14 randomized trials	not serious	not serious	not serious	not serious	none	⊕⊕⊕⊕ HIGH
Placebo/nocebo effects can have reliable effect on HRV	5 randomized trials	serious^a^	serious^b^	not serious	not serious	none	⊕⊕○○ LOW

*a. Out of six studies that investigated HRV following a placebo/nocebo effect, only two (Pollo et al., 2003; Aslaksen and Flaten, 2008) were rated with a low risk of bias. The study by Chae et al. (2008) was rated as having some concerns in risk of bias; and those of Jegindø et al. (2013), Adler-Neal et al. (2019), and De Pascalis and Scacchia (2019) were rated as having high risk of bias (see Table 3). Therefore, there are serious concerns about the current evidence. b. Heterogeneity of populations and different manipulations, small sample size, and lack of a control or a natural control history group have made some degree of inconsistency across the findings*.

## Discussion

The results of this SR disclosed the following: (1) Of eight studies on placebo and BP, four showed that there is no reliable association between placebo effect and BP, whereas four studies showed that placebo and nocebo effects are followed by lower and higher SBP, respectively. (2) Six of 14 studies on placebo and HR showed that placebo effect lowered HR, while eight studies showed that placebo effects did not impact HR. (3) Of six studies on placebo/nocebo and HRV, two showed that placebo and nocebo effects are associated with lower and higher LF-HRV, respectively. Two studies reported that HRV predicted the placebo effect, and two showed that placebo did not impact the LF/HF ratio. Only one study reported no effects of placebo on HRV (some studies had more than one result). Therefore, these results indicate that the placebo effects have reliable impact on HRV, but not on HR and BP.

### Cardiac Placebo Responses and BP

Although four studies showed that placebo analgesia and nocebo hyperalgesia were associated with lower and higher SBP, respectively, four other studies did not observe a significant change in BP following a placebo/nocebo effect.

This finding opposes the assumption of an association between placebo effects and lower SBP. All of the four studies that reported no significant relationship between placebo effect and BP used BP as an index to measure physiological stress following a placebo/nocebo effect (Robertson et al., 1991; Geers et al., 2010; Jegindø et al., 2013; Roderigo et al., 2017). Stress and negative emotions can be mediated by placebo effects; however, a placebo effect is not always accompanied with a reduction in stress. For instance, Flaten et al. (2006) showed that although positive information about a weak treatment elicited a placebo effect, it had no impact on stress level. Roderigo et al. (2017) studied the effects of stress on placebo and nocebo effects and showed that although positive information elicited lower pain, it did not impact the physiological stress level as measured by BP and HR. Therefore, a placebo effect is not necessarily associated with a lower BP.

Another reason for the lack of a placebo effect in BP is related to limitations of BP in representing cardiac SA and PA. DBP is highly affected by respiration and does not add important information about cardiac placebo responses and thus is not analyzed in most of the studies, whereas lower and higher SBP indicate higher and lower PA, respectively (Sved, 2009). Therefore, BP will not be sensitive to changes in cardiac SA following a placebo/nocebo effect. Additionally, BP is highly influenced by measurement errors such as individual (e.g., underlying hypertension history) and contextual factors (e.g., white coat hypertension effects) (Pickering et al., 2002), which partially explains the inconsistency in cardiac placebo responses in BP.

### Cardiac Placebo Responses and HR

Six studies reported an association between placebo effects and reduced HR, and eight other reported no significant association. This finding contradicts the hypothesis that there should be a reduction in HR following a placebo effect. Our previous explanation in which we suggested that a placebo response can be elicited without significantly affecting the stress level including the autonomic cardiac output (e.g., Geers et al., 2010; Bjørkedal and Flaten, 2012; Roderigo et al., 2017) is applicable here as well.

Another explanation refers to the contribution of both SA and PA on HR. This dual impact of SA and PA on HR may mask cases in which the placebo responses are more relevant to one of the autonomic branches than the other. For instance, Rhudy et al. (2018) showed that although the placebo effect modulated the skin conductance response (SCR), which is a physiological index more related to SA (Dawson et al., 2000), it did not impact HR. This finding suggests that HR failed to detect the placebo response on SA. Moreover, Schneider et al. (2007) reported that although sham acupuncture did not influence HR, it lowered cortisol levels. However, Peerdeman et al. (2015) showed that verbal suggestions did not affect either HR or the SCR.

### Cardiac Placebo Responses and HRV

Two of six studies showed that placebo effect reduced LF-HRV and that nocebo effect increased LF-HRV (Pollo et al., 2003; Chae et al., 2008). This is consistent with the hypothesis that presumes lower LF-HRV due to a placebo effect and higher LF-HRV due to a nocebo effect. Several experimental studies have reported higher LF-HRV during pain (e.g., Terkelsen et al., 2005; Pollatos et al., 2012; for a review, see Koenig et al., 2014). For instance, Aslaksen et al. (2007) showed that participants had higher LF-HRV during a heat pain task.

Therefore, contrary to the pain that increases the SA and baroreflex activity, the placebo response reduces the pain and lowers the sympathetic baroreflex activity indexed by lower LF-HRV (Pollo et al., 2003; Chae et al., 2008).

Pollo et al. (2003) showed that both the placebo effect and reduced LF-HRV remained after the muscarine antagonist atropine was administered. This finding, thus, reduces the probability of the involvement of PA in both placebo analgesia and the following cardiac placebo responses. The inverse happens for nocebo effects, in which following an increase in pain due to a nocebo treatment, the sympathetic baroreflex activity increases and leads to higher LF-HRV (Chae et al., 2008).

Two studies reported a change in LF-HRV following a placebo/nocebo effect. Reduction in LF-HRV following a placebo effect indicates decreases in SA, especially the baroreflex activity, and indicates a reduction in physiological stress. The inverse happens for a nocebo effect. Pollo et al. (2003) showed that the reduced LF-HRV following a placebo effect was reversed by the opioid antagonist naloxone and concluded that under stressful circumstances, ANS is controlled by the endogenous opioid system. However, a reduction in physiological stress is not necessarily bonded to a placebo effect, as out of six studies, only two reported such effect; this partially confirms that a placebo effect does not necessarily impact cardiac activity unless it modulates physiological stress level and negative emotions.

Two studies reported that HRV predicted the placebo effect on negative emotions and pain. Along the same line, this predictive value may be seen if the physiological stress level is modulated by placebo. Aslaksen and Flaten (2008) showed that although the placebo capsules did not impact HRV frequency, the placebo group had a lower LF/HF ratio than the control group. However, Chae et al. (2008) and De Pascalis and Scacchia (2019) reported that the placebo had no impact on the LF/HF ratio. This suggests that the LF/HF ratio is not a reliable measure for how placebo impacts cardiac SA and PA. The reliability of the LF/HF ratio has been questioned in previous studies (e.g., Eckberg, 1997). The sympathovagal balance index calculated by the LF/HF ratio has been demonstrated to be theoretically flawed (e.g., Eckberg, 1997) and not empirically supported (e.g., Billman, 2013). The most serious concern is that LF does not index SA (e.g., Houle and Billman, 1999; Goldstein et al., 2011). Thus, there is a lack of rationale and compelling evidence that the LF/HF ratio indexes the relative impact of both vagal activity and SA (e.g., Hopf et al., 1995; Reyes del Paso et al., 2013).

## Conclusion

This review investigated the effects of placebo analgesia and nocebo hyperalgesia on cardiac activity indexed by BP, HR, and HRV and shows that HRV seems as a better index to detect the effects of placebo/nocebo on cardiac activity.

Cardiac activity is dually controlled by sympathetic and parasympathetic systems, and it is not possible to track the separate effects of each autonomic branch from HR and BP recordings (Thayer and Lane, 2000; Sved, 2009). In line with previous studies (e.g., Pollo et al., 2003; Aslaksen and Flaten, 2008; Koenig et al., 2014), this SR concludes that HRV is a more reliable method to study cardiac placebo analgesic and nocebo hyperalgesic responses since this method can represent both sympathetic and parasympathetic influences on cardiac activity (Malik, 1996; Task force of the European society of cardiology the north American society of pacing electrophysiology., 1996).

Furthermore, the results of this SR confirm that the elicitation of a placebo effect is not necessarily dependent on the modulation of autonomic and physiological stress levels, since a placebo can occur without significantly affecting the physiological stress (e.g., Flaten et al., 2006). This assumption partially explains why a considerable number of studies fail to observe a cardiac placebo response (e.g., Colloca and Benedetti, 2009; Geers et al., 2010; Bjørkedal and Flaten, 2012).

## Recommendation for Prospective Studies

Firstly, to measure cardiac placebo/nocebo responses, the present SR provides evidence that BP and HR are not optimal in detecting cardiac placebo responses and therefore recommends HRV over BP and HR; however, as the quality of evidence supporting this recommendation is rated as low, more documentation is needed to underpin the advantages of HRV in placebo studies. Secondly, sex differences in cardiac activity were reported in five studies; however, only three analyzed sex differences on cardiac placebo responses (Robertson et al., 1991; Bjørkedal and Flaten, 2012; Roderigo et al., 2017) (see Table 2); two studies reported that females had lower SBP than males, but the effect of sex on cardiac placebo responses was not reported (Aslaksen et al., 2014, 2015). However, two other studies reported that participants' sex did not affect BP following a placebo effect (Robertson et al., 1991; Roderigo et al., 2017). Of three studies that analyzed sex differences in HR following a placebo effect, one reported that females' placebo responses were accompanied with a reduced HR as compared with males (Robertson et al., 1991), whereas two studies reported no sex differences on HR following a placebo (Bjørkedal and Flaten, 2012; Roderigo et al., 2017). Although Aslaksen and Flaten (2008) showed that male subjects reported higher placebo effect to male experimenters, participants' sex differences in HRV were not analyzed; and no other studies considered sex differences in HRV following a placebo/nocebo effect. Therefore, potential sex differences in cardiac placebo responses remain to be investigated by future studies. Thirdly, this SR focused on the effects of placebo analgesia and nocebo hyperalgesia on cardiac activity; however, the effects of placebo on cardiac activity in other symptoms (e.g., itch, hypertension, and fatigue) are not well-known and need more investigation. Fourth, this SR investigated cardiac placebo responses and concluded that a placebo effect does not necessarily lower physiological stress. This speculation needs to be investigated using other physiological stress measurements (SCR, endocrine secretion, electroencephalography, etc.) as well.

## Limitations

There are some limitations to this SR. Firstly, as this is a qualitative SR, generalizing findings to other fields and symptoms requires caution. Secondly, the majority of the included studies were on healthy volunteer samples. Therefore, extrapolating findings to, e.g., patients with chronic pain must be done with caution. Thirdly, due to different terms and inconsistent keywords used across studies, access to all relevant studies may have been limited. However, to ensure the inclusion of all relevant studies, we manually reviewed the citation and reference lists of all included studies, as in similar previous SRs (Daniali and Flaten, 2019). A fourth limitation is the lack of a thorough report on the method of cardiac data recording as in Aslaksen et al. (2014) or a thorough report on cardiac analysis method as in Roderigo et al. (2017), which hinders a clear conclusion. Fifthly, although HRV is recommended over BP or HR, the lower number of HRV studies than that of BP or HR studies has limited the quality of evidence supporting this notion. Finally, although this SR did not have a review protocol, the scientific nature of the study was precisely characterized by specifying *a priori* questions and a relevant review procedure.

## Data Availability Statement

Publicly available datasets were analyzed in this study. This is a review article based on published Works.

## Author Contributions

HD and MF both had significant contribution in planning the study, reviewing the articles, analyzing the results, writing the draft, and preparing the final manuscript. All authors contributed to the article and approved the submitted version.

## Conflict of Interest

The authors declare that the research was conducted in the absence of any commercial or financial relationships that could be construed as a potential conflict of interest.
